# Multiple Transient Signals in Human Visual Cortex Associated with an Elementary Decision

**DOI:** 10.1523/JNEUROSCI.3835-16.2017

**Published:** 2017-06-07

**Authors:** Thomas Meindertsma, Niels A. Kloosterman, Guido Nolte, Andreas K. Engel, Tobias H. Donner

**Affiliations:** ^1^Department of Psychology and; ^2^Amsterdam Brain and Cognition Center, University of Amsterdam, 1018 WS, Amsterdam, The Netherlands,; ^3^Department of Neurophysiology and Pathophysiology, University Medical Center Hamburg-Eppendorf, 20246 Hamburg, Germany, and; ^4^Max Planck UCL Centre for Computational Psychiatry and Ageing Research, Max Planck Institute for Human Development, 14195 Berlin, Germany

**Keywords:** beta oscillations, cortical state, perceptual decision-making, pupil-linked arousal, visual cortex

## Abstract

The cerebral cortex continuously undergoes changes in its state, which are manifested in transient modulations of the cortical power spectrum. Cortical state changes also occur at full wakefulness and during rapid cognitive acts, such as perceptual decisions. Previous studies found a global modulation of beta-band (12–30 Hz) activity in human and monkey visual cortex during an elementary visual decision: reporting the appearance or disappearance of salient visual targets surrounded by a distractor. The previous studies disentangled neither the motor action associated with behavioral report nor other secondary processes, such as arousal, from perceptual decision processing per se. Here, we used magnetoencephalography in humans to pinpoint the factors underlying the beta-band modulation. We found that disappearances of a salient target were associated with beta-band suppression, and target reappearances with beta-band enhancement. This was true for both overt behavioral reports (immediate button presses) and silent counting of the perceptual events. This finding indicates that the beta-band modulation was unrelated to the execution of the motor act associated with a behavioral report of the perceptual decision. Further, changes in pupil-linked arousal, fixational eye movements, or gamma-band responses were not necessary for the beta-band modulation. Together, our results suggest that the beta-band modulation was a top-down signal associated with the process of converting graded perceptual signals into a categorical format underlying flexible behavior. This signal may have been fed back from brain regions involved in decision processing to visual cortex, thus enforcing a “decision-consistent” cortical state.

**SIGNIFICANCE STATEMENT** Elementary visual decisions are associated with a rapid state change in visual cortex, indexed by a modulation of neural activity in the beta-frequency range. Such decisions are also followed by other events that might affect the state of visual cortex, including the motor command associated with the report of the decision, an increase in pupil-linked arousal, fixational eye movements, and fluctuations in bottom-up sensory processing. Here, we ruled out the necessity of these events for the beta-band modulation of visual cortex. We propose that the modulation reflects a decision-related state change, which is induced by the conversion of graded perceptual signals into a categorical format underlying behavior. The resulting decision signal may be fed back to visual cortex.

## Introduction

Perceptual decisions transform perceptual signals into categorical behavioral reports. Even the most elementary of such decisions entail a complex cascade of central events. Those decisions include transient activations of prefrontal association cortex (Frässle et al., 2014; Brascamp et al., 2015), top-down feedback interactions between higher-tier and lower-tier cortical regions (Lamme and Roelfsema, 2000; Nienborg and Cumming, 2009; Zagha et al., 2013; van Kerkoerle et al., 2014; Wimmer et al., 2015), transient release of neuromodulators (Aston-Jones and Cohen, 2005; Parikh et al., 2007; Lin et al., 2015), boosts of arousal state (de Gee et al., 2014; Kloosterman et al., 2015a), and changes in fixational eye movements (Bonneh et al., 2010).

Previous studies have identified a transient modulation of visual cortical population activity during behavioral reports of the following simple perceptual events: the appearance and disappearance of a salient visual target (Wilke et al., 2006, 2009, Donner et al., 2008, 2013; Maier et al., 2008; Kloosterman et al., 2015b). This modulation is widespread across the retinotopic map of visual cortex, expressed in the 12–30 Hz (beta) frequency range, evident during illusory and stimulus-evoked perceptual events, and weaker during passive viewing (Donner et al., 2008; Wilke et al., 2009; Kloosterman et al., 2015b).

We hypothesized that the beta-band modulation is a top-down signal indicating a state change of visual cortex, induced by the conversion of a graded perceptual signal into a categorical “decision state.” Critically, the previous studies characterizing the beta-band modulation in visual cortex disentangled neither the motor action used for the report nor other secondary processes from perceptual decision processing. To overcome this limitation and address the above hypothesis, we here assessed the role of the motor act and a number of other candidate processes that might affect the state of visual cortex, namely: an increase in pupil-linked arousal, fixational eye movements, and fluctuations in bottom-up sensory processing indexed by gamma-band responses.

The preparation and execution of motor movements are associated with beta-band modulations in the motor system (Donner et al., 2009; Engel and Fries, 2010), and corticocortical feedback from motor cortex influences the state of sensory cortex (Zagha et al., 2013). Here, we uncoupled the perceptual events from motor preparation and execution (O'Connell et al., 2012). The state of sensory cortex is also strongly affected by the modulatory arousal systems of the brainstem, which exhibit phasic responses during perceptual decisions (Aston-Jones and Cohen, 2005; Parikh et al., 2007; Lin et al., 2015; Varazzani et al., 2015; Joshi et al., 2016; Nelson and Mooney, 2016). We thus related pupil dilation, a peripheral marker of brainstem activity (Varazzani et al., 2015; Joshi et al., 2016), and cortical arousal state (McGinley et al., 2015) to the modulation in visual cortex during perceptual events.

We found prominent beta-band modulations in visual cortex during target disappearances and reappearances, during both silent counting and overt button presses, indicating a nonmotor origin of the modulation. This beta-band modulation was dissociated in several respects from power modulations associated with pupil-linked arousal, fixational eye movements, and fluctuations of bottom-up sensory processing that are reflected in gamma-band power.

## Materials and Methods

### Subjects

Thirty-one subjects participated in the experiment, which consisted of two experimental sessions each of ∼2 h each. Two subjects were excluded due to not completing all sessions. One further subject was excluded after the first session due to bad eye-tracking data quality. Thus, 28 subjects (17 female subjects; age range, 20–54 years; mean age, 28.3 years; SD, 9.2) were included in the analysis. All subjects had normal or corrected-to-normal vision and no known history of neurological disorders. The experiment was conducted in accordance with the Declaration of Helsinki and approved by the local ethics committee of the Hamburg Medical Association. Each subject gave written informed consent.

### Behavioral tasks and experimental design

**Figure 1.**
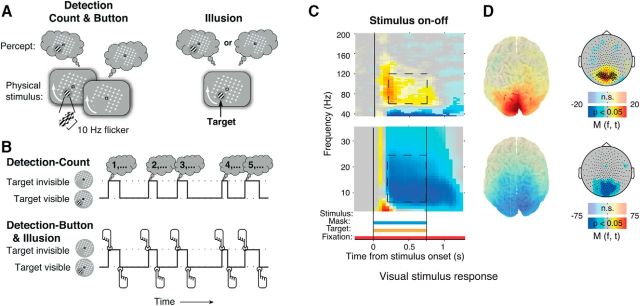
Stimuli, behavioral tasks, and stimulus response in visual cortex. ***A***, Schematic snapshots of stimuli and alternating percepts. Left, Detection stimulus. A salient, flickering target stimulus (Gabor patch) was surrounded by a moving mask pattern (white), which appeared as a rotating grid. The target was intermittently removed from the display. Top left, The corresponding perception of the target. Right, Corresponding stimulus and alternating perception during the Illusion condition, in which the target did not flicker or physically disappear. Perceptual disappearances (top right) were illusory in this condition. ***B***, Behavioral task conditions. Top, Detection-Count. Subjects counted the disappearances during the 3 min run and reported the total after the end of the run in a 4AFC question. Bottom, Detection-Button and Illusion. Subjects reported target disappearances and reappearances by alternating button presses of two buttons. ***C***, Cortical response to the stimulus during Stimulus-on-off. Fully saturated colors indicate clusters of significant modulation (*p* < 0.05, two-sided permutation test across subjects, cluster-corrected; *N* = 23 subjects). ***D***, Source maps and scalp maps, topography of 8–25 and 60–120 Hz modulations (0.25–0.75 s after stimulus onset; see dashed outlines on time–frequency representations). Highlighted circles in high-frequency scalp map (top right): MEG sensors showing the biggest stimulus response. These sensors were used for the subsequent analyses of overall power modulation.

Subjects performed several tasks based on the same basic visual stimulus (Fig. 1*A*). Subjects completed a total of 44 3 min runs, divided over blocks of 2 or 4 runs. Subjects performed the two main conditions, Detection and Illusion (see below), in different blocks, with the order of the blocks counterbalanced across subjects. The Stimulus-on-off condition (see below) was performed at the end of one of the two sessions.

#### Stimulus

The stimulus used for all conditions consisted of a salient target (full contrast Gabor patch; diameter, 2°; two cycles), which was located either in the lower left or lower right visual field quadrant (eccentricity, 5°; counterbalanced between subjects), surrounded by a rotating mask (17° × 17° grid of white crosses) and superimposed on a gray background (Fig. 1*A*). During the Detection condition (see below), the cycles of the Gabor patch modulated at opposite phase at a temporal frequency of 10 Hz. The resulting counterphase flicker rendered the target more salient, thus minimizing the number of illusory target disappearances. The mask rotated at a speed of 160°/s. The target was separated from the mask by a gray “protection zone” subtending ∼2° around the target (Bonneh et al., 2001). Subjects fixated on a fixation mark (red outline, white inside, 0.8° width and length) centered on the mask in the middle of the screen. Stimuli were presented using the Presentation Software (NeuroBehavioral Systems). Stimuli were back-projected on a transparent screen using a Sanyo PLC-XP51 projector with a resolution of 1024 × 768 pixels at 60 Hz. Subjects were seated 58 cm from the screen in a whole-head magnetoencephalography (MEG) scanner setup in a dimly lit room.

#### Detection and illusion conditions

In the main experimental conditions (Detection and Illusion), the stimulus was continuously presented for multiple runs with a duration of 3 min each. During the Detection condition, the target was physically removed from the screen at variable times and for variable durations, which were manipulated for predictability. The target disappearance durations were drawn, in different runs, from different distributions corresponding to different hazard rates (i.e., conditional probability of occurrence of target on-/offset, given that it has not yet occurred; Luce, 1986). The conditional probability distributions were one of two Gaussians (i.e., relatively predictable; μ = 2 s, σ = 0.2 s or μ = 6 s, σ = 0.6 s, respectively) or a uniform distribution (i.e., unpredictable; μ = 6 s, truncated at 14 s). The effects of the temporal statistics of target disappearances and reappearances on pupil responses are described in a separate report (Kloosterman et al., 2015a). On different runs, subjects either immediately reported the target disappearances and reappearances by button press (Detection-Button), or they silently counted the target disappearances (Detection-Count). Detection-Button or Detection-Count conditions were randomly selected on each run, under the constraint that both would occur equally often. The instructions for each condition were displayed on the screen before the run started. Subjects could only start the next run after they confirmed the instructions to the experimenter over the intercom.

In the Detection-Button runs, subjects were instructed to report target disappearances and reappearances by pressing a button with their right index finger and middle finger, respectively. The visual stimulus was identical, and target disappearance and reappearance durations were drawn from the same distributions in Detection-Count and Detection-Button runs. The only systematic difference was that Detection-Count runs did not require immediate behavioral report of the perceptual changes. Instead, subjects counted the number of target disappearances that occurred during the 3 min run and reported the total in a four-alternative forced-choice (4AFC) question after the end of the run (Fig. 1*B*) by pressing one of four buttons with their right hand. The three incorrect alternatives were generated by adding a number from the set (−3, −2, −1, 1, 2, or 3) to the true number of disappearances. These numbers were randomly selected under the constraint that the four alternatives were all different from each other. This design made it impossible for subjects to anticipate, during the Detection-Count runs, which of the four response buttons would have to be pushed at the end of the run, minimizing motor preparation.

The Illusion condition was identical to Detection-Button with the exception that the target remained physically present on the screen throughout the whole run (without flicker) and disappeared and reappeared subjectively, due to an illusion dubbed motion-induced blindness (Bonneh et al., 2001; Bonneh and Donner, 2011). As the subjects' overt behavioral reports were required to determine the timing of target disappearances and reappearances of the target, no silent counting condition was included for the Illusion condition. In the Illusion condition, subjects reported that the target was invisible for 19% of the total viewing duration (average across subjects). The distribution of the target disappearance durations had a characteristic asymmetric shape with a rapid rise and long tail, resembling a gamma distribution. The median target disappearance was 1.64 s across subjects (median of medians).

Because we used a Gabor patch as the target stimulus, the overall stimulus luminance remained constant during physical target onsets and offsets, so that retinal influences on fluctuations in pupil diameter during the task were minimized (Loewenfeld, 1993; Kloosterman et al., 2015a). Subjects completed a total of 19 runs each of the Detection-Count and Detection-Button conditions, respectively, and six runs of the Illusion condition.

#### Stimulus-on-off

During the Stimulus-on-off condition, subjects viewed the complete visual stimulus (gray background, white rotating mask, fixation, and target) for 0.75 s, preceded and succeeded by only the background and fixation. This stimulus duration was too short to induce illusory target disappearances but was sufficiently long to measure the stimulus-induced modulation of cortical population activity (Fig. 1*C*,*D*). Subjects were instructed to maintain fixation and passively view the stimulus onsets and offsets. The Stimulus-on-off condition was completed by 24 of the subjects; 1 of these subjects was excluded from the analysis of the stimulus-induced responses because of technical problems with the MEG data collection.

### MEG, eye tracking, and magnetic resonance imaging

MEG data were acquired on a 275-channel MEG system (VSM/CTF Systems) with a sample rate of 1200 Hz. Subjects were placed in a seated position inside the scanner. The location of the subjects' heads was measured in real time using three fiducial markers placed in both ears and on the nasal bridge to control for excessive movement. Furthermore, electrooculography and electrocardiography were recorded to aid artifact rejection. T1-weighted structural magnetic resonance images (MRIs) were acquired from all subjects for reconstruction of source-level activity.

Concurrently with the MEG recordings, the diameter of the pupil of the left eye was sampled at 1000 Hz with an average spatial resolution of 15–30 min arc, using an EyeLink 1000 Long Range Mount (SR Research). This MEG-compatible (nonferromagnetic) setup was placed on a table under the stimulus presentation screen. The eye tracker was calibrated before every block of four runs.

### Data analysis

The data were analyzed in MATLAB (MathWorks) using the Fieldtrip toolbox (Oostenveld et al., 2011) and custom-made software.

#### Trial extraction

For the Stimulus-on-off condition, we extracted trials of fixed durations, ranging from 0.2 s before to 0.75 s after stimulus onset.

For the Detection and Illusion conditions involving subjects' reports, we extracted trials of variable duration, centered on subjects' button presses, from the 3 min runs of continuous stimulation. In the case of the Illusion condition, the term “trial” refers to an epoch of constant stimulation and is solely defined based on subjects' subjective reports of target disappearance and reappearance. We call this method for trial extraction “response locked”. The following constraints were used to avoid mixing data segments from different percepts when averaging across trials, as follows: (1) the maximum trial duration ranged from −1.5 to 1.5 s relative to the report; (2) when another report occurred within this interval, the trial was terminated 0.5 s from this report; (3) when two reports succeeded one another within 0.5 s, no trial was defined; and (4) for the analysis of the Detection-Button condition, we included only those reports that were preceded by a physical change of the target stimulus within 0.2–1 s, thus discarding reports following illusory target disappearances.

In an alternative analysis of all Detection conditions, trials were defined in the same way as described above but now aligned to physical target onsets and offsets (“stimulus-locked”). In the Detection-Count conditions, no button responses occurred during the run, so stimulus-locked trial extraction was the only option. We used this method in every analysis that involved the Detection-Count condition.

#### Preprocessing

##### MEG data.

The following preprocessing steps were performed: trial extraction (see Trial extraction); environmental, muscle, jump and eye artifact rejection; removal of line noise; and resampling to 500 Hz. All epochs that contained artifacts caused by environmental noise, eye, muscle activity, or squid jumps were excluded from further analysis using standard automatic methods included in the Fieldtrip toolbox. Epochs that were marked as containing an artifact were discarded after every artifact detection step. For all artifact detection steps, the artifact thresholds were set individually for all subjects. Both of these choices aimed at optimization of artifact exclusion. Line noise was removed by subtracting the 50, 100, 150, and 200 Hz Fourier components from the raw MEG time course of each trial. For all trials that contained a MEG artifact, the pupil data were also discarded. Although the data quality of some of these trials was sufficient to include them in the analysis, we focused on the MEG signal and its relation to pupil diameter in this article. We therefore excluded all trials that had insufficient MEG data quality, regardless of the quality of the corresponding pupil diameter data.

##### Pupil data.

Eyeblinks were detected using the standard algorithms of the manufacturer of the eyetracker system. When a trial contained a blink that occurred within 0.5 s of the stimulus onset or offset, the trial was discarded. Blinks that were not within 0.5 s of a stimulus event were removed by linear interpolation of the values of blink onset and offset plus 0.1 s of padding. During some epochs, the pupil signal was of unacceptable quality due to difficulties specific to pupil measurements (e.g., incorrect threshold settings or partial covering of the pupil by the eyelid), while the MEG data were of sufficient quality. In these trials, the pupil data were discarded, but the trials were still used for the MEG analyses that did not include pupil data.

Pupil responses during cognitive events are sluggish and confined to a frequency range of <4 Hz (Hoeks and Levelt, 1993; Loewenfeld, 1993). Consequently, signal fluctuations of >4 Hz mainly reflect measurement noise. To remove the high-frequency noise, the pupil time series were low-pass filtered using a third-order Butterworth filter with a cutoff of 4 Hz. The filtered pupil time series was then transformed to percent signal change.

#### Spectral analysis of MEG power

We used sliding window Fourier transform (Mitra and Pesaran, 1999; step size, 50 ms; window length, 500 ms for perceptual change-related and microsaccade-related modulation or 200 ms for pupil-related modulation) to calculate time–frequency representations of the MEG power (spectrograms) for each sensor and each single trial. We used a single Hanning taper for the frequency range of 3–35 Hz (frequency resolution, 2.5 Hz; bin size, 1 Hz; frequency range for pupil-related modulation, 5–35 Hz) and the multitaper technique for the frequency range of 36–150 Hz (spectral smoothing, 8 Hz; bin size, 2 Hz; five tapers). To optimize frequency resolution, we used Welch's method to compute frequency spectra of pupil-related modulation (see Fig. 4*E*,*F*). The signal of each sensor was converted to two orthogonal planar gradients before time–frequency analysis. After time–frequency analysis, the planar gradients of each sensor were recombined by taking the sum of their power values.

For Stimulus-on-off, spectrograms were averaged aligned to stimulus onsets. For Detection and Illusion conditions, spectrograms were averaged aligned to perceptual reports of target disappearances/reappearances (Detection and Illusion) or aligned to the corresponding target offsets/onsets (Detection).

#### Trial-related modulations of MEG power

Power modulations [denoted as *M*(*f*,*t*) in all figures] during the trials were quantified as the percentage of power change at a given frequency bin and time point, relative to a “baseline” power value for each frequency bin (see next section). We subtracted the trial-specific baseline value from each sample in the time course per frequency bin and divided by the mean baseline power across all trials.

#### Baseline intervals for MEG power modulations

**Figure 2.**
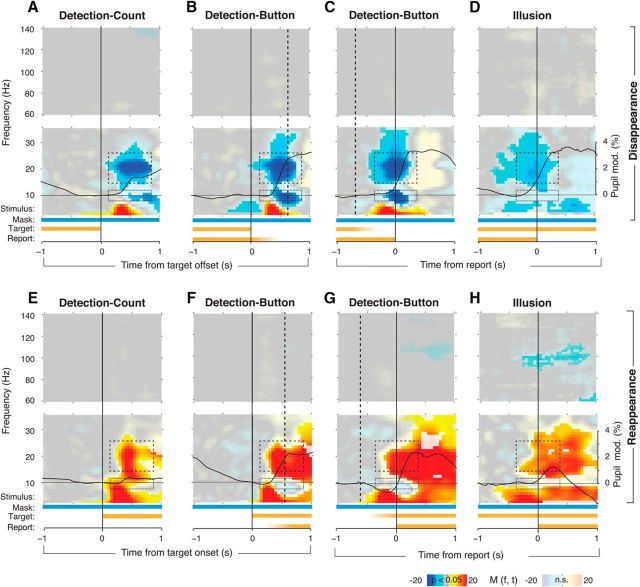
Perceptual modulation with or without motor act. MEG power modulations are shown as time–frequency representations, averaged across trials within each subject and then across subjects. Fully saturated colors highlight clusters of significant modulation (*p* < 0.05, two-sided permutation test across subjects, cluster corrected). In each panel, the top and bottom time-frequency representation correspond to the high- and low-frequency ranges, respectively. The bars underneath the time–frequency representations depict the time course of stimulus components and subjects' reports. Fading indicates variable timing of the instantaneous stimulus changes or report with respect to the trigger. Solid black time courses: pupil responses (group average). Dashed boxes indicate alpha power (8–12 Hz) and beta power (15–25 Hz) modulation. Different panels correspond to different experimental conditions and different trigger events. ***A***, Detection-Count, aligned to stimulus offset. ***B***, Detection-Button, aligned to stimulus offset. Dashed line indicates the median reaction time. ***C***, Detection-Button, aligned to disappearance report. Dashed line corresponds to the median time of stimulus offset. ***D***, Illusion, aligned to the disappearance report. ***E–H***, Corresponding modulation aligned to stimulus onset or reappearance report.

For Stimulus-on-off, the baseline was computed as the mean power across the prestimulus blank fixation interval (from −0.25 to 0 s relative to stimulus onset). For the Detection and Illusion conditions, the baseline was computed in the following two different ways: (1) the mean power across the whole trial interval and across all trials, as in an earlier study (Kloosterman et al., 2015b); or (2) the single-trial power averaged across a pre-event (i.e., pre-response or pre-stimulus change for response-locked and stimulus-locked analyses, respectively) time window. For the latter version, the time windows ranged from −1.25 to −0.75 s for response-locked and −1 to −0.5 s for stimulus-locked analyses, respectively. For simplicity, the trial average baseline version 1 is not shown here. As expected, it revealed differences in power modulation levels before the experimental events of interest, which were eliminated, by construction, in version 2. Critically, however, both versions yielded qualitatively identical transient post-event responses. We used the pre-event, single-trial baseline (version 2) for all analyses reported in this article, because this version specifically isolated the transient power modulation in response to the perceptual event, and it was identical to the approach used for quantifying transient pupil dilation responses to the perceptual events (see next subsection; Fig. 2; see also Fig. 5).

We focused our analysis of MEG power modulation around perceptual reports on those cortical regions that also processed the physical stimulus (i.e., visual cortex). Therefore, before performing the statistical tests described in this and the following section, we averaged all power modulation across the 25 occipital sensors exhibiting the biggest stimulus-induced high-frequency response (60–120 Hz) during Stimulus-on-off (Fig. 1*D*). See the study by Kloosterman et al. (2015b) for a similar procedure.

#### Modulation of MEG power related to pupil responses

Evoked pupil responses were computed as the difference between the baseline pupil diameter and the peak of the evoked pupil response time course during the trial. Pupil dilation responses typically peak at ∼1 s after the triggering event (Hoeks and Levelt, 1993; de Gee et al., 2014). Therefore, we detected the response peak in the time window from 0.5 to 2 s from the stimulus change. From this peak, we subtracted the pupil baseline diameter in the window directly before (from 0.25 to 0.5 s after stimulus change). This window was chosen to rule out the possibility that pupil-related effects in the MEG power are caused by ongoing fluctuations in the pupil signal that are not related to the task. This approach resulted in one scalar value of transient pupil dilation per trial.

To examine the relationship between MEG power modulation and pupil response, we sorted the MEG data (pooled across all conditions) into three bins (equal number of trials) based on the pupil response amplitude: low, medium, and high transient pupil dilation. This was done separately for disappearance and reappearance trials. We focused on stimulus-locked analysis for this characterization, because this allowed us to include both the Detection-Button and Detection-Count conditions. However, similar results were found for response-locked analyses, also when we included only the Illusion condition (data not shown).

Pupil responses were negatively correlated with pupil baseline (Kloosterman et al., 2015a, their Supplementary Fig. 2). Accordingly, the low, medium, and high pupil response bins also differed in terms of baseline pupil diameter. Our focus was on the relation between transient modulations of MEG power modulation and the evoked pupil responses. Thus, we corrected for differences in pupil baseline between pupil response bins, referred to as “pupil response condition” in the following sections. For this procedure, we used only the data from the low and high pupil response conditions. Separately for both conditions, we further sorted trials by their baseline pupil diameter values into 10 equally spaced bins. This yielded two distributions of baseline pupil diameters, one for each pupil response condition. The aim of our procedure was to match these two distributions as closely as possible. To this end, we randomly discarded trials from the pupil response condition with a higher number of trials until the number of trials was equalized. This procedure was repeated separately for each baseline pupil bin. For example, if baseline pupil bin number 1 contained 15 trials of the low pupil dilation condition and 18 trials of the high pupil dilation condition, three randomly selected trials from the latter condition were discarded. The procedure was repeated 200 times to minimize random biases. We averaged over all iterations of the procedure.

A second possible confound in our analyses was the duration since the last stimulus change. Because the perceptual changes in this study were either caused by or mimicked spontaneous illusory perceptual changes, the variability in duration since the last change was high. It is possible that for some trials the transient modulations in pupil size and/or the MEG power modulation did not completely return to baseline before the next perceptual change, thereby affecting the signals on the subsequent trial. We accounted for these effects in two ways. First, we excluded trials that followed the previous trial within 1 s. Second, we removed the component of power modulations that was explained by previous trial duration (via linear regression) from the pupil dilation scalar values and the power in every time–frequency bin from −1.5 to 1.5 s from stimulus change. This analysis step did not qualitatively change the results (data not shown).

#### Control for microsaccades and microsaccade-related modulation of MEG power

To control for the effects of changes in residual fixational eye movements (i.e., those remaining after rejecting trials with large eye movements, see above) around perceptual changes (Bonneh et al., 2010), we detected microsaccades using a previously established algorithm (Engbert and Kliegl, 2003; Engbert and Mergenthaler, 2006; Dimigen et al., 2011; http://www2.hu-berlin.de/eyetracking-eeg) and the same parameters as in previous studies (Bonneh et al., 2010; Kloosterman et al., 2015b). The binary time course of microsaccade occurrences was convolved with a Gaussian window (σ = 0.1 s) to compute a continuous estimate of microsaccade rate. The trial-to-trial change in the microsaccade rate was quantified as the number of microsaccades during the time window showing the MEG modulations (0.15–0.75 s after stimulus change) minus the number of microsaccades in the preceding window (−0.45 to 0.15 s with respect to stimulus change). This difference was then correlated with single-trial modulations of MEG power. To also characterize the MEG power modulations regardless of microsaccades, we performed selective analyses of only those trials without any microsaccades in the time window 0.15–0.75 s after stimulus change.

#### Control for beta-gamma power correlations and stimulus-evoked responses

We performed two analyses to rule out the effects of variations in the strength of bottom sensory responses on the beta-band modulations characterized here. First, we quantified the trial-to-trial correlation between power modulations in beta bands (15–25 Hz) and gamma bands (60–120 Hz) from 0.25 to 0.75 s after stimulus change. We then removed (via linear regression) the component of beta-power trial-to-trial fluctuations that was explained by gamma-band power to verify that the residual beta-band signal still exhibited a robust modulation during perceptual events.

Second, we assessed the role of evoked sensory responses to the flickering target during the Detection conditions in the generation of the beta-band modulations. To this end, we subtracted the time-domain average (a measure of the phase-locked, stimulus-evoked response) from the single-trial signals before spectral analysis. This isolated the non-phase-locked (“induced”) modulations of MEG power (Donner and Siegel, 2011). This analysis yielded power modulations (data not shown) that were qualitatively indistinguishable from the modulations of total power shown in Results. Furthermore, we observed a robust beta-band modulation also in the Illusion condition (see Results), in which the target did not flicker. Together, these observations rule out the concern that flicker-evoked sensory processes were responsible for the beta-band modulation. For simplicity, we quantified the total power modulations (i.e., the sum of phase-locked and non-phase-locked modulations) for all analyses throughout this article.

#### Source reconstruction of MEG power modulations

We used a variant of an adaptive spatial filtering technique called linear beamforming (Van Veen et al., 1997; Gross et al., 2001) for source reconstruction of power modulations. In short, for each frequency and source location, a linear filter was computed that passes activity from that location with unit gain while maximally suppressing activity from other sources. The source-level analyses were performed independently for the frequency bands of 16–24 Hz for perceptual modulation and of 9–15 Hz for pupil-related modulation. We used the measured head positions and individual single-shell volume conductor models, based on individual images from T1-weighted structural MRI, to compute a common spatial filter over the transient window of interest and a baseline window. This was performed separately for every MEG recording run because the orientation of the subjects' heads with respect to the MEG sensors was not necessarily comparable between measurements. The transient intervals that were used for the perceptual modulation were 0.25–0.75 s from stimulus change or −0.25 to 0.25 from response for stimulus-locked and response-locked analyses, and 0.1–0.7 s from stimulus change for the pupil-related modulation. The baseline windows were −0.5 to 0 s from stimulus change, −1 to −0.5 s from response, and −1 to −0.4 s from stimulus change, respectively. For each MEG recording run, source-level transient power modulation was computed by projecting the sensor-level data from the transient window through the common spatial filter, and subtracting and dividing by the baseline data that was projected to the same filter. The resulting source-level modulation maps were nonlinearly aligned to a template brain (Montreal Neurological Institute) using the individual images from structural MRI.

### Statistical tests

**Figure 3.**
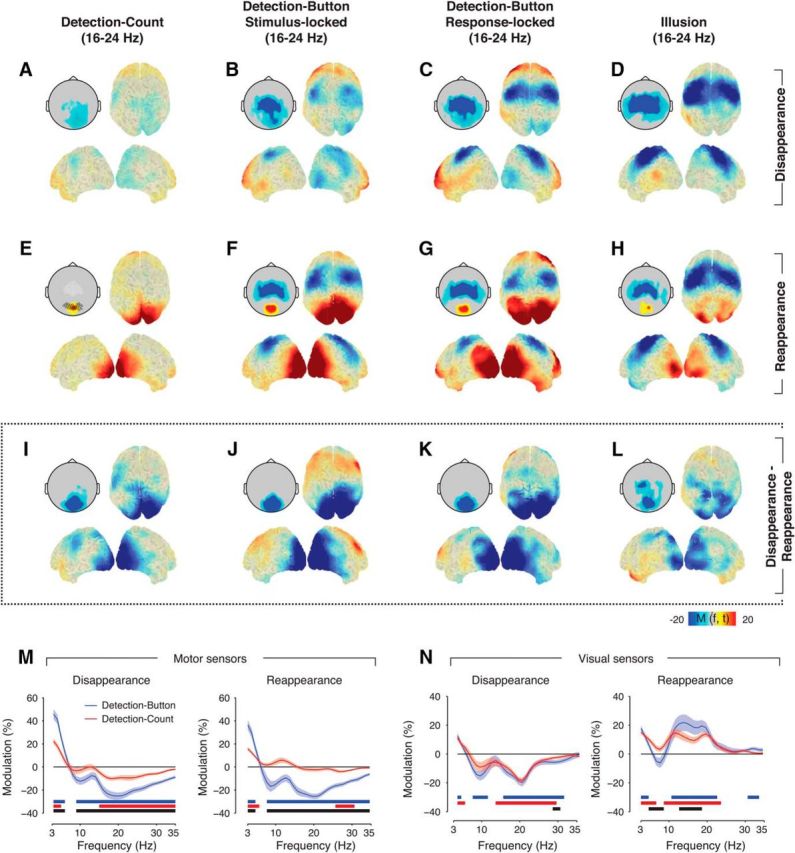
Dissociation of perceptual and movement-related modulation. Source reconstructions and topographical scalp maps of 16–24 Hz modulations around target disappearance, reappearance, and the difference for all conditions. The highlighted black circles on the scalp map in ***E*** indicate occipital sensors that were used for the time–frequency representations in Figure 2 and frequency spectra in ***N***, highlighted white circles indicate motor sensors used in ***M***. ***A***, Detection-Count, disappearance. ***B***, Detection-Button disappearance, locked to stimulus offset. ***C***, Detection-Button disappearance, locked to report of disappearance. ***D***, Illusion disappearance. ***E–H***, Corresponding maps for target reappearance. ***I–L***, Corresponding maps for disappearance − reappearance difference. ***M***, Spectra of the power modulation in sensors overlying motor cortex (white circles in ***E***) of the Detection-Button (blue) and Detection-Count condition (red) in the time window around median response time (0.45–0.75 s from stimulus change), separately for disappearance (left) and reappearance trials (right). Shaded areas correspond to SEM over subjects. Solid bars under the spectra reflect clusters of significant power modulation (*p* < 0.05, two-sided permutation across subjects, cluster corrected) for Detection-Button (blue), Detection-Count (red), and the difference between Detection-Button and Detection-Count (black). ***N***, Corresponding results for visually responsive sensors as defined in Figure 1*D* (black circles in *E*).

**Figure 4.**
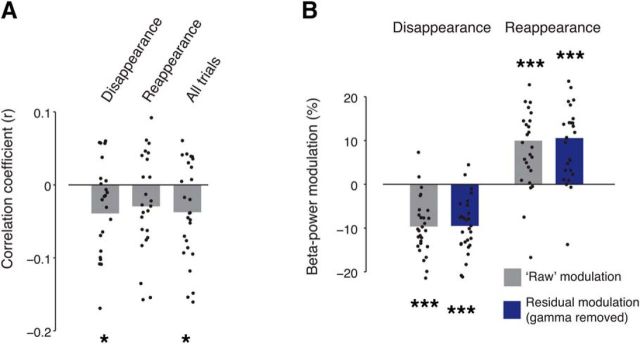
Fluctuations in gamma-band power do not account for perceptual modulation. ***A***, Trial-to-trial correlation between transient beta-band (15–25 Hz) and gamma-band (60–120 Hz) power in transient time window (0.25–0.75 s after stimulus change) across subjects, for disappearance trials, reappearance trials, or both conditions combined (“All trials”). Group average; dots depict individual subjects (*p < 0.05, two-sided permutation test across subjects, 10,000 permutations). ***B***, Average beta-band modulation before (gray bars) and after (blue bars) removing trial-to-trial fluctuations of gamma-band power (***p < 0.001, two-sided permutation test across subjects, 10,000 permutations).

**Figure 5.**
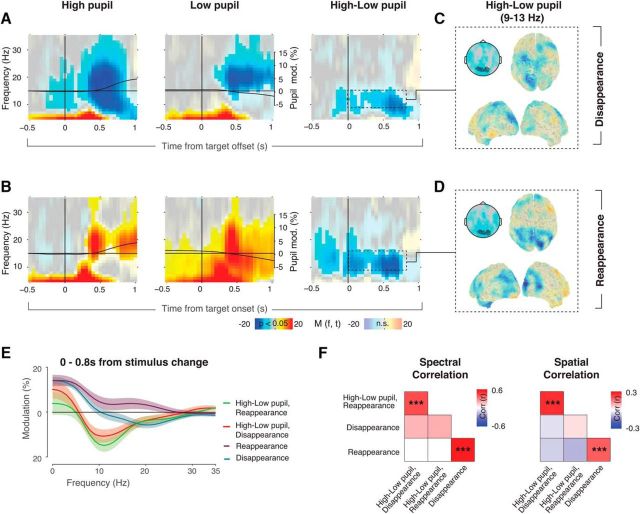
Distinct signatures of perceptual and pupil-linked modulations in visual cortex. ***A***, Time–frequency representation of visual cortex power around target offsets, for trials with high (left) and low phasic pupil dilation (middle), and the difference (right). Fully saturated colors highlight clusters of significant modulation (*p* < 0.05, two-sided permutation test across subjects, cluster corrected). The solid black lines indicate time courses of pupil diameter, averaged over subjects. ***B***, Corresponding results for target reappearance. ***C***, Source reconstructed maps and scalp maps of the difference between high and low pupil dilation in the window indicated by the dashed box in ***A***. ***D***, Corresponding results for target reappearance. ***E***, Frequency spectra of the modulation in the time window of 0–0.8 s with respect to stimulus change, compared with prestimulus change baseline. Shaded area corresponds to the SEM over subjects. ***F***, Correlation matrices between the pupil-related and perceptual modulations of the spectra (left matrix, based on spectra shown in *E*) and spatial topographies (right matrix). Pearson correlations were performed per subject and were tested for significance using a permutation test (10,000 permutations, FDR corrected for multiple comparisons, ***p < 0.001).

We used a two-tailed permutation test (1000 permutations; Efron and Tibshirani, 1994) to test the significance of the overall power modulation of the sensor group obtained in Stimulus-on-off. To quantify switch-related modulations, we tested the overall power modulation for significant deviations from zero. For pupil-related modulations, we tested the overall power modulation for (1) significant deviations from zero per pupil condition and (2) significant differences between the high pupil dilation and low pupil dilation condition. For all these tests, we used a cluster-based procedure (Maris and Oostenveld, 2007) to correct for multiple comparisons. For time–frequency representations of power modulation (Figs. 1*C*, 2, 4*A*,*B*, and 5), this procedure was conducted across all time-frequency bins; for spectra of power modulations (Fig. 3*M*,*N*), this procedure was performed across frequencies. For statistical tests of correlations, we first computed correlation coefficients within subjects and then tested the individual correlation coefficients against zero across subjects using permutation tests (corrected for multiple comparisons using a false discovery rate correction at α = 0.05). This was done to assess the trial-to-trial power correlations between beta and gamma bands (see above) as well as the spectral and spatial pattern correlations (i.e., across frequency bins or sensors) of power modulations (see Fig. 5*F*).

## Results

We analyzed modulations of MEG power that occur around salient, behaviorally relevant perceptual events: the disappearances and reappearances of a full-contrast target (Gabor patch) surrounded by a moving mask (Fig. 1). In different conditions, target disappearances and reappearances were either evoked by the physical offsets and onsets of the target or they occurred subjectively due to an illusion called motion-induced blindness (Bonneh et al., 2001; Bonneh and Donner, 2011). To characterize modulations of cortical population activity in visual cortex during the main experimental conditions (Detection and Illusion), we selected the 25 sensors that exhibited the strongest stimulus-induced increase in 60–140 Hz (gamma) power during Stimulus-on-off (Fig. 1*C*, top, dashed box; see also Materials and Methods). All selected sensors were located over the occipital cortex (Fig. 1*D*, top, topographic plot, circles), and the gamma-band responses peaked in bilateral visual cortices (Fig. 1*D*).

To assess the impact of the preparation or execution of motor movement on the modulations in visual cortical activity around the perceptual events, we asked subjects to silently count the target disappearances and report the total at the end of the run, with a motor response that was unpredictable during the perceptual events (see Materials and Methods). We also analyzed transient modulations of visual cortical activity as a function of phasic arousal related to perceptual events. We here operationalized “phasic arousal” as evoked pupil responses. This operational definition was based on recent animal work, which established remarkably strong correlations between non-luminance-mediated variations in pupil diameter and global cortical arousal state (McGinley et al., 2015).

### Top-down modulation of beta-band activity in visual cortex in the absence of motor act

We observed similar modulations of MEG activity over visual cortex around perceptual events in all three conditions, Detection-Button, Detection-Count, and Illusion (Figs. 2, 3). MEG power decreased in the beta band (∼15–25 Hz) in visual cortex around target disappearance during all conditions (Figs. 2*A–D*, top, dashed boxes, 3*A–D*), peaking around the time of behavioral report, or ∼500 ms after target offset during Detection-Count, corresponding to the median response time in Detection-Button. Around target reappearance, power in the same beta frequency band increased (Fig. 2*E–H*, top, dashed boxes, 3*E–H*). This pattern of post-event modulations of MEG power was robust with respect to the baseline interval used for computing the power modulations (see Materials and Methods).

Additional power modulations were evident in the theta (4–8 Hz) and alpha (∼10 Hz) frequency bands, but those were more variable across conditions (Fig. 2, bottom dashed boxes). For example, robust alpha-band modulations were only observed during the Detection conditions and were absent during the Illusion condition. In all conditions, the transient power modulations spared out the gamma band (Fig. 2), which exhibited robust stimulus-induced modulations during Stimulus-on-off (Fig. 1*C*).

Power modulations after physical target offsets and onsets (in the Detection conditions) might be related to the changes in retinal input associated with these localized perceptual events confined to the position of the target. However, retinal input was constant during the Illusion condition, indicating that the beta-band modulation common to all conditions was more closely associated with the subjective perceptual events than bottom-up sensory processing. Furthermore, as expected based on previous fMRI results (Donner et al., 2008), source reconstruction showed that the beta-band modulations were more widespread across occipital and parietal cortex than expected from the localized perceptual events (Fig. 3).

To further test for a relation to fluctuations in bottom-up processing of the whole stimulus (i.e., target and mask), we used trial-to-trial variations in gamma-band power, which, on average, was robustly driven by the stimulus (Fig. 1*C*). We correlated power modulations in the beta and gamma band to each other (see Materials and Methods). Although, on average, the gamma-band response during the perceptual events was negligible (Fig. 2), there was substantial trial-to-trial variability in gamma-power response. These trial-to-trial fluctuations exhibited small, but significant, negative correlations with the trial-to-trial fluctuations of the beta-band modulations for both target disappearances and all trials combined (Fig. 4*A*). However, both the beta-power suppression after disappearance and the beta-power enhancement after reappearance were not affected by removing (via linear regression) the component of the trial-to-trial fluctuations shared with gamma power (Fig. 4*B*). If anything, the residual modulations were even stronger.

Together, the results indicated that the beta-band modulation reflected a nonsensory modulation of the global state of visual cortex. Subtracting the beta-power modulations around target disappearance and reappearance yielded a single measure of the (differential) modulation reflecting the binary behavioral report. In the following, we refer to this differential signal as the “perceptual modulation” and show that it was not affected by motor act, pupil-linked arousal, or fixational eye movements.

### Perceptual modulation of visual cortex in the absence of motor act

Across conditions, the perceptual modulation peaked in visual cortex with little modulation in motor cortices (Fig. 3*I–L*). The latter is due to the fact that motor cortices exhibited similar modulation (movement-related beta-band suppression; Donner et al., 2009) during reports of both target disappearance and reappearance, which is in line with previous observations (Kloosterman et al., 2015b; Fig. 3*B–D*,*F–H*).

The design of the Detection-Count condition made it impossible for subjects to know when a response was required and which button had to be pressed. However, it is possible that subjects covertly prepared motor responses during Detection-Count, as they did in the Button condition, without executing them. To assess this possibility, we analyzed beta-band suppression over motor cortex (Fig. 3*E*, white circles), a reliable marker of motor preparation and execution (Donner et al., 2009; Engel and Fries, 2010). As expected, we found strong beta-band suppression around the median response time (from 0.45 to 0.75 s after stimulus change) in the Detection-Button condition after both target disappearance and reappearance (Fig. 3*M*). In the Detection-Count condition, beta-band suppression was significantly weaker after target disappearance and was completely absent after reappearance. These beta-band modulations over motor cortex were in stark contrast to the beta-band modulations in visual cortex, where we found robust modulations for both the Detection-Button and Detection-Count conditions (Figs. 2, 3*A–L, N*, which is inconsistent with the idea that motor preparation was the driving factor of the beta-band modulation in visual cortex.

In summary, robust beta-band modulations around perceptual changes occurred in visual cortex both during immediate behavioral report (Detection-Button and Illusion) and silent counting without motor report (Detection-Count). The near absence of robust beta-band suppression in motor cortex in Detection-Count suggests that the role of motor preparation in the beta-band modulation in visual cortex was negligible. Together, these findings indicated that a considerable component of the transient beta-band modulation around the perceptual changes is unrelated to motor processing.

### Disentangling perceptual and pupil-linked modulations in visual cortex

Brainstem neuromodulatory systems regulating central arousal state, in particular the noradrenergic locus ceruleus and the cholinergic basal forebrain, are phasically activated during elementary perceptual decisions (Aston-Jones and Cohen, 2005; Parikh et al., 2007; Lin et al., 2015). We tracked such phasic boosts in central arousal by measuring pupil dilation around the perceptual events (Einhäuser et al., 2008; de Gee et al., 2014; Kloosterman et al., 2015a; McGinley et al., 2015; Varazzani et al., 2015; Joshi et al., 2016).

Across all experimental conditions, the pupil dilated during both target disappearance and reappearance (Fig. 2, black overlaid time courses). This invariant positive sign of the pupil modulations for target disappearance and reappearance stood in contrast to the opposite sign of the MEG power modulations (Fig. 2; see also Donner et al., 2008; Kloosterman et al., 2015b). Thus, pupil dilation and the beta-band modulation exhibited, on average, distinct functional behavior.

To examine the relationship between pupil-linked neuromodulatory responses and the MEG in more detail, we sorted trials based on the amplitude of pupil responses, into Low, Medium, and High pupil response bins and compared the associated power modulations in visual cortex (Fig. 5; see Materials and Methods). The pupil responses exhibited a large trial-to-trial variability. High pupil trials were accompanied by pupil dilation, whereas Low pupil trials exhibited pupil constriction on average. This was true for both target disappearance (Fig. 5*A*, black overlaid time courses) and reappearance (Fig. 5*B*).

The beta-band modulation in visual cortex around perceptual events was of the same sign for Low and High pupil trials (i.e., negative or positive, depending on the type of perceptual event; Fig. 5*A*,*B*). But, critically, there was no robust difference in beta-power modulation between the High and Low pupil trials (Fig. 5*A*,*B*, right column). By contrast, pupil dilation did affect power modulations in the alpha band, which is consistent with recent measurements in mouse visual cortex (Reimer et al., 2014; see Discussion). Target disappearances were accompanied by stronger alpha-band suppression for High compared with Low pupil trials (Fig. 5*A*, right). Strikingly, the same was found for target reappearance (Fig. 5*B*, right), even though the overall power modulation during reappearance was positive across the entire low-frequency range (Fig. 5*B*, left, middle). Notably, for both perceptual events the difference in the pupil-related modulation in the alpha band already started before the stimulus change, suggesting an endogenous origin of this modulation. The topography of the pupil-related alpha-band modulation was more widespread (Fig. 5*C*,*D*) than that of the beta-power modulation around perceptual events (compare Figs. 3*A–L*, 5*C*,*D*).

We also characterized the relationship between pupil-linked power modulations and those related to perceptual events by quantifying their similarity in the spectral and the spatial domains. To this end, we correlated the spectra and spatial maps of the perceptual modulation (difference between disappearance and reappearance) and the pupil-linked modulation (difference between High and Low pupil trials). For the spatial domain, we used the topographies of the Detection-Count condition only, as the correlation in the Detection-Button condition would be conflated with the movement-related power modulations over motor cortex (Fig. 3*B–D*,*F–H*). We found no robust correlations between the perceptual and pupil-related power modulations in either the spectral (Fig. 5*E*) or the spatial domains (Fig. 5F, bottom left of correlation matrices). By contrast, there were robust correlations between High and Low pupil trials (Fig. 5*F*, top left of correlation matrices) and between target disappearance and reappearance (Fig. 5*F*, matrices, bottom right), indicating that these correlation analyses were sufficiently sensitive to pick up robust effects. These findings provided further support for the notion that the perceptual and pupil-linked modulations were due to distinct underlying mechanisms.

### Beta-band modulation in visual cortex not due to changes in retinal illumination or fixational eye movements

Because early visual cortex responds to transient changes in luminance (Rossi et al., 1996; Haynes et al., 2004), one might wonder whether the modulations in visual cortical activity might be merely due to changes in retinal illumination, mediated by pupil dilation and constriction. A number of observations ruled out this concern. First, the retinal illumination scenario predicted cortical responses that succeed pupil dilation by a latency corresponding to the retino–geniculo–cortical transmission delay. By contrast, even the only robust pupil-linked modulation observed here, the alpha-band suppression, occurred earlier, already reaching significance before the start of pupil dilation (Fig. 5*A*,*B*, compare modulations in right column to pupil time courses in left and middle columns). Second, the association between beta-band modulation and pupil responses was overall weak (see previous section). Third, retinal illumination predicts a power modulation in the same direction for both target disappearances and reappearances (pupil dilated during both; Figs. 2, 5) and in opposite directions for pupil dilation and constriction. Both of these predictions were in sharp contrast to the observed beta-band modulation: as described above, the beta-band suppression was still robust in trials with pupil constriction (Fig. 5*A*,*B*). Finally, modulations in cortical activity due to variations in luminance seem to be confined to early visual cortex (Yellin et al., 2015), in contrast to the widespread distribution of perceptual (Fig. 3*I–L*) and pupil-linked (Fig. 5*C*,*D*) power modulations across extrastriate visual and parietal cortex that we observed here.

**Figure 6.**
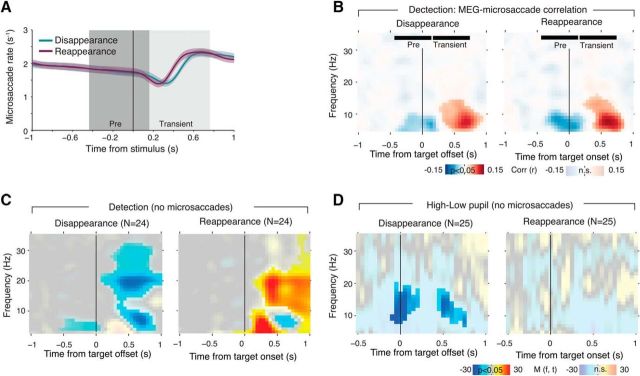
Perceptual modulation is not due to change in the microsaccade rate. ***A***, Microsaccade rate around stimulus change. Solid lines reflect the average microsaccade rate over subjects, shaded areas correspond to SEM over subjects. The dark (Pre) and light gray (Transient) areas depict the intervals used to compute the transient microsaccade rate modulation. ***B***, Time–frequency representation of the correlation between transient modulation of microsaccade rate and MEG power modulation. Fully saturated colors highlight clusters of significant modulation (*p* < 0.05, two-sided permutation test across subjects, cluster corrected). The “Pre” and “Transient” time windows correspond to the intervals used to compute the microsaccade rate modulation (see ***A***). ***C***, Time–frequency representation of transient power modulation around target disappearance and reappearance, including only those trials in which no microsaccades were detected. Fully saturated colors highlight clusters of significant modulation (*p* < 0.05, two-sided permutation test across subjects, cluster corrected). ***D***, Corresponding analysis of high and low pupil response.

Another concern might be that systematic changes in fixational eye movements around the time of target disappearance or reappearance (Bonneh et al., 2010) might have evoked modulations of visual cortical activity, for example, due to the ensuing retinal transients. We have used the current MEG dataset to rule out this concern for the Illusion condition in an earlier report focusing on the neural basis of the perceptual dynamics in motion-induced blindness (Kloosterman et al., 2015b, their Fig. 7). In the present study, we ran the same control for the Detection conditions. Consistent with earlier findings, we found a decrease in microsaccades after both target disappearance and reappearance (Fig. 6*A*; Bonneh et al., 2010).

This change in microsaccade rate was positively correlated (on a trial-by-trial basis) with modulations of low-frequency MEG power over visual cortex after target onsets or offsets. This correlation was significant from the lowest frequency bin resolved (3 Hz) up to ∼12 Hz, and it peaked in a theta range at ∼7 Hz (Fig. 6*B*). This microsaccade-related modulation of theta-band power might have been related to previous observations from local field potential recordings in monkey visual cortex (Bosman et al., 2009).

The time–frequency representation of the correlation between MEG power and change in microsaccade rate in Figure 6*B* was distinct from the spectra of perceptual modulations in Figure 3. The significant correlation in Figure 6*B* was uniformly positive after the perceptual events, whereas the modulations in Figure 2, *B* and *F*, comprised both positive (for frequencies <8 Hz) and negative modulations (for alpha and beta bands), respectively. Critically, the significant correlation in Figure 6*B* did not overlap with the perception-related beta-band modulation in Figure 2, *B* and *F*. Further, when selectively analyzing perceptual modulations, only those trials that did not contain any detected microsaccades around the perceptual events (see Materials and Methods), we found the same modulations of alpha and beta power as in Figure 2, *B* and *F*, except for the absence of a significant positive modulation <8 Hz (Fig. 6*C*).

Because the correlation between changes in microsaccade rates and MEG power in Figure 6*B* overlapped with the pupil-linked modulations in Figure 5, *A* and *B*, we repeated the analysis of pupil-linked modulations selectively on trials without detected microsaccades around perceptual events. As expected, effects were overall weaker than in Figure 5*A*. But, critically, we again found a relative suppression of alpha-band power around target disappearance for high versus low pupil dilation (Fig. 6*D*). Together, the analyses presented in this section provide strong evidence that the modulation of visual cortical beta band related to perception was not due to microsaccades.

## Discussion

The cerebral cortex continuously undergoes changes in its internal state. Cortical state changes occur during the transition from sleep or anesthesia to wakefulness (Steriade, 2000; Harris and Thiele, 2011; Haider et al., 2013). Mounting evidence shows that cortical state changes also occur rapidly during full wakefulness, then often coupled to cognitive acts like decisions (Ress and Heeger, 2003; Aston-Jones and Cohen, 2005; Jack et al., 2006; Wilke et al., 2006, 2009; Parikh et al., 2007; Donner et al., 2008; Harris and Thiele, 2011; Choe et al., 2014; Kloosterman et al., 2015b; McGinley et al., 2015). These latter state changes may originate from a variety of sources, including feedback from motor cortical processing (Zagha et al., 2013), phasic arousal (Aston-Jones and Cohen, 2005; Parikh et al., 2007), or fixational eye movements (Bonneh et al., 2010).

We here established that task-relevant disappearances and reappearances of salient visual targets were associated with a rapid state change in visual cortex, indexed by modulations of cortical population activity in the beta band. This was true regardless of whether the perceptual events were evoked by the physical stimulus or induced by a perceptual illusion (i.e., during constant sensory input). Using a silent counting condition without motor act enabled us to here dissociate this beta-band modulation from motor factors. We also dissociated the beta-band modulation from pupil-linked phasic arousal, the associated changes in retinal illumination, microsaccades, and gamma power-indexed fluctuations in bottom-up processing (Donner and Siegel, 2011).

The one factor that was consistently associated with the beta-band modulation was the behavioral relevance of the perceptual changes. Both the beta-power suppression during target disappearance and the beta-power enhancement during target reappearance were robust when perceptual changes had to be reported directly (during Illusion or Detection-Button) or when they were needed for delayed report of the total disappearance count after the end of the block (during Detection-Count; Figs. 2*A*,*B*, 3*N*). During Detection-Count, target onsets did not have to be counted explicitly, but we assume they nonetheless underwent the same decision transformation as that occurring during Detection-Button (see next paragraph) to facilitate the registration of the disappearances that had to be counted. Previous studies found that the beta-power suppression during target disappearance was weakened during passive viewing (Wilke et al., 2009; see Replay-passive in Kloosterman et al., 2015b). The results from these previous studies are consistent with the notion that behavioral relevance of the perceptual changes is critical for the beta-band modulations, but the design of the previous studies did not disentangle the following two processes related to behavioral relevance: decision processing and motor action. By disentangling these two processes, the present study established that the beta-band modulation is specifically associated with the conversion from a perceptual into a behavioral format but not with the motor act per se.

We propose that the beta-band modulation in visual cortex is a top-down feedback signal from decision-related brain regions converting graded perceptual signals into categorical behavioral reports (Nienborg and Cumming, 2009; Wimmer et al., 2015). The present task required the binary report of the disappearance (and then continued absence) or reappearance (and then continued presence) of the target. Thus, we assume that brain regions involved in decision processing transformed graded (and fluctuating) visual cortical responses to the target into a binary decision signal. This decision signal attained a high level for reappearance and a low level for disappearance, thereby driving the binary (immediate or delayed) behavioral reports. The signal was fed back to visual cortex (Nienborg and Cumming, 2009; Wimmer et al., 2015), modulating power in the beta band, which explained the bimodal nature of the modulation for target reappearance and disappearance. The decision feedback might originate from higher cortical areas (Gold and Shadlen, 2007; Siegel et al., 2011) or from subcortical nuclei (e.g., the pulvinar of the thalamus; Wilke et al., 2009), and it may help enforce a coherent decision state across cortical processing stages (Stocker and Simoncelli, 2008).

While our interpretation accounts for the bimodal nature of the beta-power modulations during the tasks studied here, some aspects of the data suggest a more complex picture. First, the beta-band modulations were transiently pronounced around the perceptual changes and then decayed back toward a lower level (of the same sign as the preceding transient) during the target-visible and target-invisible periods. Possibly, the decision signal that was being fed back exhibited similar transient and sustained components. Second, the beta-band modulation was retinotopically global and not confined to the subregions in visual cortex corresponding retinotopically to the small target (Fig. 3; Donner et al., 2008; Kloosterman et al., 2015b). This aspect argues against an underlying feedback mechanism akin to top-down selective attention, which modulates visual cortical population activity in a retinotopically specific fashion (Siegel et al., 2008; Gregoriou et al., 2009). Third, one previous observation also points to qualitative differences between the beta-band modulations during target disappearances and reappearances: the beta-enhancement after target reappearance remained evident during passive viewing (Kloosterman et al., 2015b). Thus, the beta suppression after disappearance might have reflected a purely top-down signal, as outlined above, whereas the beta enhancement after reappearance might also have contained a bottom-up component. Future work could address this idea by diverting attention away from the target disappearances and reappearances.

The ∼15–25 Hz range of the top-down signal, sometimes referred to as “beta 1,” has been implicated in integrative modes of cortical processing (Donner and Siegel, 2011; Womelsdorf et al., 2014). Beta-band activity in visual cortex can be decoupled from spiking activity (Wilke et al., 2006) and visual stimulus properties (Belitski et al., 2008). Modulations of beta-band activity reflect perceptual suppression in the pulvinar nucleus of the thalamus (Wilke et al., 2009) and near-threshold detection in visual and frontoparietal association cortex (Donner et al., 2007). The modulations might reflect neuromodulatory input (Belitski et al., 2008; Safaai et al., 2015), large-scale network reverberation (Donner et al., 2007; Siegel et al., 2011), and/or feedback interactions from higher-tier to visual cortical areas (Bastos et al., 2012, 2015; Siegel et al., 2012).

Phasic increases in pupil-linked arousal during perceptual events were, although robustly present, not essential for the beta-band modulation to occur. However, phasic pupil-linked arousal robustly influenced visual cortical alpha-band activity. Pupil dilation is associated with various cognitive processes, such as learning (Nassar et al., 2012), decision-making and uncertainty (de Gee et al., 2014; Lempert et al., 2015; Urai et al., 2017), orienting (Wang and Munoz, 2015), or changes in perception (Einhäuser et al., 2008; Hupé et al., 2009; Kloosterman et al., 2015a).

Recent work has established a close link between pupil diameter and cortical state, which may be the neurophysiological basis of all of the above phenomena. In particular, our findings are in line with recent work in rodents showing that neuronal membrane potentials in cortex are depolarized and their low-frequency (2–10 Hz) power is suppressed during periods of pupil dilation (Reimer et al., 2014) or locomotion, an effect dependent on noradrenaline (Polack et al., 2013). A similar suppression of low-frequency power occurs in visual cortical local field potentials in visual cortex during pupil dilation, regardless of whether the dilation coincides with locomotion (Vinck et al., 2015). In addition, noise correlations in visual and somatosensory cortex decrease during pupil dilation (Reimer et al., 2014). The pupil-related alpha-band suppression we observed might have been a large-scale correlate of these effects observed at the microscales and mesoscales.

The current findings ruled out pupil-linked arousal as a prerequisite of the top-down signal in visual cortex, but they did not exclude the possibility that phasic neuromodulatory input unrelated to pupil diameter might be involved in the beta-band modulations reported here. A number of studies have shown that pupil dilation robustly coincides with activation of the locus ceruleus (Murphy et al., 2014; Varazzani et al., 2015; Joshi et al., 2016; Reimer et al., 2016), making noradrenaline an unlikely candidate. Phasic release of acetylcholine from the basal forebrain is a possible candidate, as this system has been found to play a role in sensory detection and cholinergic inputs from the basal forebrain that can modulate activity in early visual cortex, even in the absence of visual stimulation (Parikh et al., 2007; Fu et al., 2014). However, recent evidence has established a cholinergic contribution to non-luminance-mediated pupil dynamics (Nelson and Mooney, 2016; Reimer et al., 2016). Noncholinergic influences of the basal forebrain on cortex might also be involved (Lin et al., 2015), and the role of the serotonergic system (Lottem et al., 2016) in transient cognitive acts and visual cortical state is largely unexplored.

In sum, our current results provide support for the idea that the transformation of perceptual events into a categorical format available for behavioral report induces a rapid state change in human visual cortex. Visual cortex is an adaptive processor, the state of which is constantly sculpted by ongoing changes in task demands and cognitive state. Beta-band activity might be a fingerprint of these ongoing adjustments in state.

## References

[B1] Aston-Jones G, Cohen JD (2005) An integrative theory of locus coeruleus-norepinephrine function: adaptive gain and optimal performance. Annu Rev Neurosci 28:403–450. 10.1146/annurev.neuro.28.061604.135709 16022602

[B2] Bastos AM, Usrey WM, Adams RA, Mangun GR, Fries P, Friston KJ (2012) Perspective canonical microcircuits for predictive coding. Neuron 76:695–711. 10.1016/j.neuron.2012.10.038 23177956 PMC3777738

[B3] Bastos AM, Vezoli J, Bosman CA, Schoffelen JM, Oostenveld R, Dowdall JR, De Weerd P, Kennedy H, Fries P (2015) Visual areas exert feedforward and feedback influences through distinct frequency channels article visual areas exert feedforward and feedback influences through distinct frequency channels. Neuron 85:390–401. 10.1016/j.neuron.2014.12.018 25556836

[B4] Belitski A, Gretton A, Magri C, Murayama Y, Montemurro MA, Logothetis NK, Panzeri S (2008) Low-frequency local field potentials and spikes in primary visual cortex convey independent visual information. J Neurosci 28:5696–5709. 10.1523/JNEUROSCI.0009-08.2008 18509031 PMC6670798

[B5] Bonneh Y, Donner T (2011) Motion induced blindness. Scholarpedia 6:3321 10.4249/scholarpedia.3321

[B6] Bonneh YS, Cooperman A, Sagi D (2001) Motion-induced blindness in normal observers. Nature 411:798–801. 10.1038/35081073 11459058

[B7] Bonneh YS, Donner TH, Sagi D, Fried M, Cooperman A, Heeger DJ, Arieli A (2010) Motion-induced blindness and microsaccades: cause and effect. J Vis 10(14):22, 1–15. 10.1167/10.14.22 21172899 PMC3075454

[B8] Bosman CA, Womelsdorf T, Desimone R, Fries P (2009) A microsaccadic rhythm modulates gamma-band synchronization and behavior. J Neurosci 29:9471–9480. 10.1523/JNEUROSCI.1193-09.2009 19641110 PMC6666524

[B9] Brascamp J, Blake R, Knapen T (2015) Negligible fronto-parietal BOLD activity accompanying unreportable switches in bistable perception. Nat Neurosci 18:1672–1678. 10.1038/nn.4130 26436901 PMC4603386

[B10] Choe KW, Blake R, Lee SH (2014) Dissociation between neural signatures of stimulus and choice in population activity of human V1 during perceptual decision-making. J Neurosci 34:2725–2743. 10.1523/JNEUROSCI.1606-13.2014 24523561 PMC3921435

[B11] de Gee JW, Knapen T, Donner TH (2014) Decision-related pupil dilation reflects upcoming choice and individual bias. Proc Natl Acad Sci U S A 111:E618–E625. 10.1073/pnas.1317557111 24449874 PMC3918830

[B12] Dimigen O, Sommer W, Hohlfeld A, Jacobs AM, Kliegl R (2011) Co-registration of eye movements and EEG in natural reading: analyses and review. J Exp Psychol Gen 140:552–572. 10.1037/a0023885 21744985

[B13] Donner TH, Siegel M (2011) A framework for local cortical oscillation patterns. Trends Cogn Sci 15:191–199. 10.1016/j.tics.2011.03.007 21481630

[B14] Donner TH, Siegel M, Oostenveld R, Fries P, Bauer M, Engel AK (2007) Population activity in the human dorsal pathway predicts the accuracy of visual motion detection. J Neurophysiol 98:345–359. 10.1152/jn.01141.2006 17493916

[B15] Donner TH, Sagi D, Bonneh YS, Heeger DJ (2008) Opposite neural signatures of motion-induced blindness in human dorsal and ventral visual cortex. J Neurosci 28:10298–10310. 10.1523/JNEUROSCI.2371-08.2008 18842889 PMC2570589

[B16] Donner TH, Siegel M, Fries P, Engel AK (2009) Buildup of choice-predictive activity in human motor cortex during perceptual decision making. Curr Biol 19:1581–1585. 10.1016/j.cub.2009.07.066 19747828

[B17] Donner TH, Sagi D, Bonneh YS, Heeger DJ (2013) Retinotopic patterns of correlated fluctuations in visual cortex reflect the dynamics of spontaneous perceptual suppression. J Neurosci 33:2188–2198. 10.1523/JNEUROSCI.3388-12.2013 23365254 PMC3608931

[B18] Efron B, Tibshirani RJ (1998) An introduction to the bootstrap. Boca Raton, FL: Chapman and Hall/CRC.

[B19] Einhäuser W, Stout J, Koch C, Carter O (2008) Pupil dilation reflects perceptual selection and predicts subsequent stability in perceptual rivalry. Proc Natl Acad Sci U S A 105:1704–1709. 10.1073/pnas.0707727105 18250340 PMC2234208

[B20] Engbert R, Kliegl R (2003) Microsaccades uncover the orientation of covert attention. Vision Res 43:1035–1045. 10.1016/S0042-6989(03)00084-1 12676246

[B21] Engbert R, Mergenthaler K (2006) Microsaccades are triggered by low retinal image slip. Proc Natl Acad Sci U S A 103:7192–7197. 10.1073/pnas.0509557103 16632611 PMC1459039

[B22] Engel AK, Fries P (2010) Beta-band oscillations-signalling the status quo? Curr Opin Neurobiol 20:156–165. 10.1016/j.conb.2010.02.015 20359884

[B23] Frässle S, Sommer J, Jansen A, Naber M, Einhäuser W (2014) Binocular rivalry: frontal activity relates to introspection and action but not to perception. J Neurosci 34:1738–1747. 10.1523/JNEUROSCI.4403-13.2014 24478356 PMC6827584

[B24] Fu Y, Tucciarone JM, Espinosa JS, Sheng N, Darcy DP, Nicoll RA, Huang ZJ, Stryker MP (2014) A cortical circuit for gain control by behavioral state. Cell 156:1139–1152. 10.1016/j.cell.2014.01.050 24630718 PMC4041382

[B25] Gold JI, Shadlen MN (2007) The neural basis of decision making. Annu Rev Neurosci 30:535–574. 10.1146/annurev.neuro.29.051605.113038 17600525

[B26] Gregoriou GG, Gotts SJ, Zhou H, Desimone R (2009) High-frequency, long-range coupling between prefrontal and visual cortex during attention. Science 324:1207–1210. 10.1126/science.1171402 19478185 PMC2849291

[B27] Gross J, Kujala J, Hamalainen M, Timmermann L, Schnitzler A, Salmelin R (2001) Dynamic imaging of coherent sources: studying neural interactions in the human brain. Proc Natl Acad Sci U S A 98:694–699. 10.1073/pnas.98.2.694 11209067 PMC14650

[B28] Haider B, Häusser M, Carandini M (2013) Inhibition dominates sensory responses in the awake cortex. Nature 493:97–100. 10.1038/nature11665 23172139 PMC3537822

[B29] Harris KD, Thiele A (2011) Cortical state and attention. Nat Rev Neurosci 12:509–523. 10.1038/nrn3084 21829219 PMC3324821

[B30] Haynes JD, Lotto RB, Rees G (2004) Responses of human visual cortex to uniform surfaces. Proc Natl Acad Sci U S A 101:4286–4291. 10.1073/pnas.0307948101 15010538 PMC384733

[B31] Hoeks B, Levelt WJM (1993) Pupillary dilation as a measure of attention: a quantitative system analysis. Behav Res Methods Instrum Comput 25:16–26. 10.3758/BF03204445

[B32] Hupé J, Lamirel C, Lorenceau J (2009) Pupil dynamics during bistable motion perception. J Vis 9(7):10, 1–19. 10.1167/9.7.10 19761325

[B33] Jack AI, Shulman GL, Snyder AZ, McAvoy M, Corbetta M (2006) Separate modulations of human V1 associated with spatial attention and task structure. Neuron 51:135–147. 10.1016/j.neuron.2006.06.003 16815338

[B34] Joshi S, Li Y, Kalwani RM, Gold JI (2016) Relationships between pupil diameter and neuronal activity in the locus coeruleus, colliculi, and cingulate cortex article relationships between pupil diameter and neuronal activity in the locus coeruleus, colliculi, and cingulate cortex. Neuron 89:221–234. 10.1016/j.neuron.2015.11.028 26711118 PMC4707070

[B35] Kloosterman NA, Meindertsma T, van Loon AM, Lamme VA, Bonneh YS, Donner TH (2015a) Pupil size tracks perceptual content and surprise. Eur J Neurosci 41:1068–1078. 10.1111/ejn.12859 25754528

[B36] Kloosterman NA, Meindertsma T, Hillebrand A, van Dijk BW, Lamme VA, Donner TH (2015b) Top-down modulation in human visual cortex predicts the stability of a perceptual illusion. J Neurophysiol 113:1063–1076. 10.1152/jn.00338.2014 25411458 PMC4329440

[B37] Lamme VA, Roelfsema PR (2000) The distinct modes of vision offered by feedforward and recurrent processing. Trends Neurosci 23:571–579. 10.1016/S0166-2236(00)01657-X 11074267

[B38] Lempert KM, Chen YL, Fleming SM (2015) Relating pupil dilation and metacognitive confidence during auditory decision-making. PLoS One 10:e0126588. 10.1371/journal.pone.0126588 25950839 PMC4423945

[B39] Lin SC, Brown RE, Hussain Shuler MG, Petersen CC, Kepecs A (2015) Optogenetic dissection of the basal forebrain neuromodulatory control of cortical activation, plasticity, and cognition. J Neurosci 35:13896–13903. 10.1523/JNEUROSCI.2590-15.2015 26468190 PMC4604228

[B40] Loewenfeld IE (1993) The pupil: anatomy, physiology, and clinical applications, Ed 1 Detroit: Wayne State UP.

[B41] Lottem E, Lörincz ML, Mainen ZF (2016) Optogenetic activation of dorsal raphe serotonin neurons rapidly inhibits spontaneous but not odor-evoked activity in olfactory cortex. J Neurosci 36:7–18. 10.1523/JNEUROSCI.3008-15.2016 26740645 PMC6601795

[B42] Luce RD (1986) Response times: their role in inferring elementary mental organization, Ed 1 New York: Oxford UP

[B43] Maier A, Wilke M, Aura C, Zhu C, Ye FQ, Leopold DA (2008) Divergence of fMRI and neural signals in V1 during perceptual suppression in the awake monkey. Nat Neurosci 11:1193–1200. 10.1038/nn.2173 18711393 PMC2754054

[B44] Maris E, Oostenveld R (2007) Nonparametric statistical testing of EEG- and MEG-data. J Neurosci Methods 164:177–190. 10.1016/j.jneumeth.2007.03.024 17517438

[B45] McGinley MJ, Vinck M, Reimer J, Batista-Brito R, Zagha E, Cadwell CR, Tolias AS, Cardin JA, McCormick DA (2015) Waking state: rapid variations modulate neural and behavioral responses. Neuron 87:1143–1161. 10.1016/j.neuron.2015.09.012 26402600 PMC4718218

[B46] Mitra PP, Pesaran B (1999) Analysis of dynamic brain imaging data. Biophys J 76:691–708. 10.1016/S0006-3495(99)77236-X 9929474 PMC1300074

[B47] Murphy PR, O'Connell RG, O'Sullivan M, Robertson IH, Balsters JH (2014) Pupil diameter covaries with BOLD activity in human locus coeruleus. Hum Brain Mapp 35:4140–4154. 10.1002/hbm.22466 24510607 PMC6869043

[B48] Nassar MR, Rumsey KM, Wilson RC, Parikh K, Heasly B, Gold JI (2012) Rational regulation of learning dynamics by pupil-linked arousal systems. Nat Neurosci 15:1040–1046. 10.1038/nn.3130 22660479 PMC3386464

[B49] Nelson A, Mooney R (2016) The basal forebrain and motor cortex provide convergent yet distinct movement-related inputs to the auditory cortex. Neuron 90:635–648. 10.1016/j.neuron.2016.03.031 27112494 PMC4866808

[B50] Nienborg H, Cumming BG (2009) Decision-related activity in sensory neurons reflects more than a neuron's causal effect. Nature 459:89–92. 10.1038/nature07821 19270683 PMC2917918

[B51] O'Connell RG, Dockree PM, Kelly SP (2012) A supramodal accumulation-to-bound signal that determines perceptual decisions in humans. Nat Neurosci 15:1729–1735. 10.1038/nn.3248 23103963

[B52] Oostenveld R, Fries P, Maris E, Schoffelen JM (2011) FieldTrip: open source software for advanced analysis of MEG, EEG, and invasive electrophysiological data. Comput Intell Neurosci 2011:156869. 10.1155/2011/156869 21253357 PMC3021840

[B53] Parikh V, Kozak R, Martinez V, Sarter M (2007) Prefrontal acetylcholine release controls cue detection on multiple timescales. Neuron 56:141–154. 10.1016/j.neuron.2007.08.025 17920021 PMC2084212

[B54] Polack PO, Friedman J, Golshani P (2013) Cellular mechanisms of brain state-dependent gain modulation in visual cortex. Nat Neurosci 16:1331–1339. 10.1038/nn.3464 23872595 PMC3786578

[B55] Reimer J, Froudarakis E, Cadwell CR, Yatsenko D, Denfield GH, Tolias AS (2014) Pupil fluctuations track fast switching of cortical states during quiet wakefulness. Neuron 84:355–362. 10.1016/j.neuron.2014.09.033 25374359 PMC4323337

[B56] Reimer J, McGinley MJ, Liu Y, Rodenkirch C, Wang Q, McCormick DA, Tolias AS (2016) Pupil fluctuations track rapid changes in adrenergic and cholinergic activity in cortex. Nat Commun 7:13289. 10.1038/ncomms13289 27824036 PMC5105162

[B57] Ress D, Heeger DJ (2003) Neuronal correlates of perception in early visual cortex. Nat Neurosci 6:414–420. 10.1038/nn1024 12627164 PMC2278238

[B58] Rossi AF, Rittenhouse CD, Paradiso MA (1996) The representation of brightness in primary visual cortex. Science 273:1104–1107. 10.1126/science.273.5278.1104 8688096

[B59] Safaai H, Neves R, Eschenko O, Logothetis NK, Panzeri S (2015) Modeling the effect of locus coeruleus firing on cortical state dynamics and single-trial sensory processing. Proc Natl Acad Sci U S A 112:12834–12839. 10.1073/pnas.1516539112 26417078 PMC4611622

[B60] Siegel M, Donner TH, Oostenveld R, Fries P, Engel AK (2008) Neuronal synchronization along the dorsal visual pathway reflects the focus of spatial attention. Neuron 60:709–719. 10.1016/j.neuron.2008.09.010 19038226

[B61] Siegel M, Engel AK, Donner TH (2011) Cortical network dynamics of perceptual decision-making in the human brain. Front Hum Neurosci 5:21. 10.3389/fnhum.2011.00021 21427777 PMC3047300

[B62] Siegel M, Donner TH, Engel AK (2012) Spectral fingerprints of large-scale neuronal interactions. Nat Rev Neurosci 13:121–134. 10.1038/nrn3137 22233726

[B63] Steriade M (2000) Corticothalamic resonance, states of vigilance and mentation. Neuroscience 101:243–276. 10.1016/S0306-4522(00)00353-5 11074149

[B64] Stocker AA, Simoncelli EP (2008) A Bayesian model of conditioned perception. Adv Neural Inf Process Syst 20:1409–1416. PMC4199208 25328364

[B65] Urai AE, Braun A, Donner TH (2017) Pupil-linked arousal is driven by decision uncertainty and alters serial choice bias. Nat Commun 8:14637. 10.1038/ncomms14637 28256514 PMC5337963

[B66] van Kerkoerle T, Self MW, Dagnino B, Gariel-Mathis MA, Poort J, van der Togt C, Roelfsema PR (2014) Alpha and gamma oscillations characterize feedback and feedforward processing in monkey visual cortex. Proc Natl Acad Sci U S A 111:14332–14341. 10.1073/pnas.1402773111 25205811 PMC4210002

[B67] Van Veen BD, van Drongelen W, Yuchtman M, Suzuki A (1997) Localization of brain electrical activity via linearly constrained minimum variance spatial filtering. IEEE Trans Biomed Eng 44:867–880. 10.1109/10.623056 9282479

[B68] Varazzani C, San-Galli A, Gilardeau S, Bouret S (2015) Noradrenaline and dopamine neurons in the reward/effort trade-off: a direct electrophysiological comparison in behaving monkeys. J Neurosci 35:7866–7877. 10.1523/JNEUROSCI.0454-15.2015 25995472 PMC6795183

[B69] Vinck M, Batista-Brito R, Knoblich U, Cardin JA (2015) Arousal and locomotion make distinct contributions to cortical activity patterns and visual encoding. Neuron 86:740–754. 10.1016/j.neuron.2015.03.028 25892300 PMC4425590

[B70] Wang CA, Munoz DP (2015) A circuit for pupil orienting responses: implications for cognitive modulation of pupil size. Curr Opin Neurobiol 33:134–140. 10.1016/j.conb.2015.03.018 25863645

[B71] Wilke M, Logothetis NK, Leopold DA (2006) Local field potential reflects perceptual suppression in monkey visual cortex. Proc Natl Acad Sci U S A 103:17507–17512. 10.1073/pnas.0604673103 17088545 PMC1859959

[B72] Wilke M, Mueller KM, Leopold DA (2009) Neural activity in the visual thalamus reflects perceptual supression. Proc Natl Acad Sci U S A 106:9465–9470. 10.1073/pnas.0900714106 19458249 PMC2684842

[B73] Wimmer K, Compte A, Roxin A, Peixoto D, Renart A, de la Rocha J (2015) Sensory integration dynamics in a hierarchical network explains choice probabilities in cortical area MT. Nat Commun 6:6177. 10.1038/ncomms7177 25649611 PMC4347303

[B74] Womelsdorf T, Valiante TA, Sahin NT, Miller KJ, Tiesinga P (2014) Dynamic circuit motifs underlying rhythmic gain control, gating and integration. Nat Neurosci 17:1031–1039. 10.1038/nn.3764 25065440

[B75] Yellin D, Berkovich-Ohana A, Malach R (2015) Coupling between pupil fluctuations and resting-state fMRI uncovers a slow build-up of antagonistic responses in the human cortex. Neuroimage 106:414–427. 10.1016/j.neuroimage.2014.11.034 25463449

[B76] Zagha E, Casale AE, Sachdev RN, McGinley MJ, McCormick DA (2013) Motor cortex feedback influences sensory processing by modulating network state. Neuron 79:567–578. 10.1016/j.neuron.2013.06.008 23850595 PMC3742632

